# Characterization of Foodborne Strains of *Staphylococcus aureus* by Shotgun Proteomics: Functional Networks, Virulence Factors and Species-Specific Peptide Biomarkers

**DOI:** 10.3389/fmicb.2017.02458

**Published:** 2017-12-11

**Authors:** Mónica Carrera, Karola Böhme, José M. Gallardo, Jorge Barros-Velázquez, Benito Cañas, Pilar Calo-Mata

**Affiliations:** ^1^Department of Food Technology, Spanish National Research Council, Marine Research Institute, Vigo, Spain; ^2^Department of Analytical Chemistry, Nutrition and Food Science, School of Veterinary Sciences, University of Santiago de Compostela, Lugo, Spain; ^3^Department of Analytical Chemistry, Complutense University of Madrid, Madrid, Spain

**Keywords:** foodborne, *Staphylococcus aureus*, shotgun proteomics, mass spectrometry, functional networks, virulence factors, peptide biomarkers

## Abstract

In the present work, we applied a shotgun proteomics approach for the fast and easy characterization of 20 different foodborne strains of *Staphylococcus aureus* (*S. aureus*), one of the most recognized foodborne pathogenic bacteria. A total of 644 non-redundant proteins were identified and analyzed via an easy and rapid protein sample preparation procedure. The results allowed the differentiation of several proteome datasets from the different strains (common, accessory, and unique datasets), which were used to determine relevant functional pathways and differentiate the strains into different Euclidean hierarchical clusters. Moreover, a predicted protein-protein interaction network of the foodborne *S. aureus* strains was created. The whole confidence network contains 77 nodes and 769 interactions. Most of the identified proteins were surface-associated proteins that were related to pathways and networks of energy, lipid metabolism and virulence. Twenty-seven virulence factors were identified, and most of them corresponded to autolysins, N-acetylmuramoyl-L-alanine amidases, phenol-soluble modulins, extracellular fibrinogen-binding proteins and virulence factor EsxA. Potential species-specific peptide biomarkers were screened. Twenty-one species-specific peptide biomarkers, belonging to eight different proteins (nickel-ABC transporter, N-acetylmuramoyl-L-alanine amidase, autolysin, clumping factor A, gram-positive signal peptide YSIRK, cysteine protease/staphopain, transcriptional regulator MarR, and transcriptional regulator Sar-A), were proposed to identify *S. aureus*. These results constitute the first major dataset of peptides and proteins of foodborne *S. aureus* strains. This repository may be useful for further studies, for the development of new therapeutic treatments for *S. aureus* food intoxications and for microbial source-tracking in foodstuffs.

## Introduction

*Staphylococcus aureus* is a ubiquitous gram-positive pathogenic microorganism that can trigger a wide spectrum of human diseases, from benign skin and soft tissue infections to life-threatening diseases including bacteremia, endocarditis and pneumonia (Lowry, 1998). Its resistance to environmental stress and antimicrobial agents makes it a worldwide problem for healthcare facilities, causing nosocomial and community-acquired infections (Archer, 1998). In 2015, the World Health Organization estimated that *S. aureus* causes >350,000 illnesses/year worldwide (World Health Organization, 2015).

*Staphylococcus aureus* is also considered one of the major foodborne pathogens that can elicit serious food intoxications, mainly resulting from the contamination of food by *S. aureus*-produced enterotoxins (Hennekinne et al., 2012). These heat-stable toxins of 25–30 kDa present similar amino acid sequences and common functional properties (Krakauer and Stiles, 2003; Lindsay and Griffiths, 2013). In addition, staphylococcal pathogenicity is driven by a wide variety of virulence factors, such as autolysins, coagulases, lipases and fibrinogen-binding proteins (Zecconi and Scali, 2013). These virulence factors are involved in colonization and spreading within hosts. The general symptoms of staphylococcal food poisoning include nausea, abdominal cramping, vomiting and diarrhea (Hennekinne et al., 2012). The disease typically resolves within 24–48 h; however severe symptoms that require hospitalization can occur when infants, the elderly or debilitated people are affected.

New proteome-wide functional networks can generate interesting opportunities to discover the mode of action of relevant functional pathways, virulence factors and potential antimicrobial mechanisms of *S. aureus* (Cherkasov et al., 2011). Protein-protein interaction network studies have been performed on methicillin-resistant *S. aureus* strains (Cherkasov et al., 2011) and *S. aureus-*mediated mastitis (Ibeagha-Awemu et al., 2010). Other reports have studied the cell wall-associated proteins of four strains of *S. aureus* (Dreisbach et al., 2011) and the *in-silico* secretome of 15 strains of *S. aureus* (Khoon and Neela, 2010; Kusch and Engelmann, 2014). Nevertheless, the great potential of the current shotgun proteomics and functional bioinformatics methodologies still has not been applied to the study of foodborne strains of *S. aureus*.

Staphylococcal foodborne infections are mainly associated with improper hygienic food handling of raw, cooked and processed foods, either in the food industry or at home. European Regulation No. 852/2004 ensures that the correct hygienic practices for all foodstuffs are followed (European Regulation, 2004). The European Food Safety Authority thus recommends the development of rapid and sensitive methods for the detection of pathogenic microorganisms in foodstuffs.

Identification of *S. aureus* has been traditionally based on the typical morphology of β-hemolytic and yellow-pigmented colonies on blood agar and on phenotypic tests, including the production of coagulase, fermentation of mannitol and trehalose, and production of heat-stable nucleases (Boerlin et al., 2003). However, the preparation of routine culture procedures for *S. aureus* identification is a time-consuming strategy. It is consequently crucial to develop rapid and sensitive methods to detect the presence of *S. aureus* in foodstuffs.

In recent years, new molecular microbial diagnostic methods based on genomics and proteomics methodologies have been developed (Gallardo et al., 2013; Carrera et al., 2016). Molecular detection of *S. aureus* based on 16S rDNA genes allows the detection of several staphylococcal species (Rohrer et al., 2001; Larsen et al., 2008). However, these DNA-based techniques are time-consuming procedures that require more than a day to be completed (Rohrer et al., 2001). Recent developments in the application of proteomics for analyzing microorganisms have generated effective and rapid proteomics fingerprints for microbial identification (Demirev and Fenselauz, 2008). The use of matrix-assisted laser desorption/ionization-time-of-flight mass spectrometry (MALDI-TOF MS) analysis on intact bacterial strains has been successfully used for the accurate classification of microorganisms in foodstuffs in just a few hours (Mazzeo et al., 2006; Böhme et al., 2010, 2012). The new public database Spectrabank (http://www.spectrabank.org), contains the mass spectral fingerprints of 120 species of spoilage and pathogenic bacteria from seafood, including three strains of *S. aureus*. Moreover, advanced proteomics methods established for liquid chromatography-tandem mass spectrometry (LC-MS/MS) analysis and targeted proteomics methods based on the monitoring of specific peptide biomarkers provide the ability to detect several microbial markers in foodstuffs (Karlsson et al., 2015). In addition, specific bioinformatics analyses were performed for the *in-silico* characterization of potential drug target proteins for methicillin/vancomycin-*S. aureus* infections (Haag et al., 2015). However, a limited number of peptide biomarkers have been published for the identification of foodborne bacteria (Chenau et al., 2014). In fact, the selection of species-specific peptide biomarkers for the discrimination of foodborne *S. aureus* strains has not been explored.

In this paper, we present for the first time the characterization of 20 different foodborne strains of *S. aureus* using a shotgun proteomics approach. To accomplish this analysis, we used an easy and rapid protein sample preparation procedure to create a reference proteome dataset for 20 foodborne *S. aureus* strains in the first step. The reference proteome was then subjected to several functional bioinformatics analyses such as (i) functional pathways, gene ontology (GO) and hierarchical clustering analysis, (ii) functional protein network analysis, (iii) finding virulence factors, and (iv) the screening of potential species-specific peptide biomarkers for the discrimination of foodborne *S. aureus*.

## Materials and methods

### *Staphylococcus aureus* strains

A total of 20 different *S. aureus* strains were used in this study (Table 1). The strains were obtained from the Spanish Type Culture Collection and from the Institute of Science of Food Production of the National Research Council of Italy and were previously characterized by MALDI-TOF mass spectrometry (Böhme et al., 2012). Most of these strains are from food origins; however, strain U17 is a clinical strain. Culture collection strains ATCC 9144 and ATCC 29213 are classified as *S. aureus* subsp. *aureus*, while strain ATCC 35845 is classified as *S. aureus* subsp. *anaerobius* (http://www.cect.org/busqb.php?val1=908&orig=1&limit=100000). In previous studies, species identification of *S. aureus*, as well as the presence of several enterotoxins, was confirmed by multiplex polymerase chain reactions (multiplex PCRs) (Morandi et al., 2007; Giebel et al., 2010). All bacterial strains have been reactivated in brain heart infusion (BHI, Oxoid Ltd., Hampshire, UK) and incubated at 31°C for 24 h. Bacterial cultures were then grown on plate count agar (PCA, Oxoid) at 31°C for 24 h.

**Table 1:** *Staphylococcus aureus* (SA) strains used in this study.

**Sample**	**Strain**	**Source**	**Enterotoxins**
S1	SA_41	Cheese/Goat	A
S2	SA_92	Cheese/Cow	D
S3	SA_280	Raw milk/Cow	A, D
S4	SA_286	Raw milk/Cow	N/A
S5	SA_507	Raw milk/Cow	D
S6	SA_587	Raw milk/Cow	D
S7	SA_617	Raw milk/Cow	N/A
S8	SA_640	Raw milk/Cow	A, D
S9	SA_700	Raw milk/Cow	D
S10	SA_844	Raw milk/Cow	A, D
S11	SA_894	Raw milk/Cow	C
S12	SA_ATCC9144	CECT 59/ATCC9144	-
S13	SA_ATCC29213	CECT 794/ATCC29213	A, D, E
S14	SA_ATCC35845	CECT 4521/ATCC35845	-
S15	SA_CA19	Cheese/Goat	A
S16	SA_GP2	Raw milk/Cow	N/A
S17	SA_GP17	Raw milk/Cow	D
S18	SA_OV8	Raw milk/Sheep	D, A
S19	SA_PE1	Raw milk/Sheep	A
S20	SA_U17	Human	C

*N/A, not available*.

Although the optimal growth temperature for *S. aureus* is 37°C, the incubation was conducted at 31°C. This difference was because we applied a protocol that had been standardized in our laboratory for the analysis of unknown strains isolated from food by mass spectrometry (Böhme et al., 2012). In general, bacterial strains with optimal growth temperatures at 37°C grow well at 31°C, including *S. aureus* strains, the objective of the present study. Broth tubes were inoculated under aerobic conditions.

### Protein extraction

Protein extracts were prepared as described previously (Böhme et al., 2010; Carrera et al., 2016). Briefly, a 1 μL loop full of each bacterial culture was harvested, placed in 100 μL of a solution containing 50% acetonitrile (50% ACN) (Merck, Darmstadt, Germany) and 1% aqueous trifluoroacetic acid (1% TFA) (Acros Organics, NJ, USA) and mixed by vortexing. After the sample was centrifuged at 8,000 rpm for 10 min, the supernatant was treated with a solution of lysis buffer (60 mM Tris-HCl pH 7.5, 1% lauryl maltoside, 5 mM phenylmethanesulfonyl fluoride (5 mM PMSF) and 1% dithiothreitol (1% DTT)). After several shakes and vortexing with glass beads for 10 min at 4°C, the supernatants were obtained by centrifugation for 10 min at 40,000 × g (J221-M centrifuge, Beckman, CA, US), transferred to a new tube and then quantified using the bicinchoninic acid method (Sigma Chemical Co., US). All analyses were performed in triplicate.

### Peptide sample preparation

Protein extracts were subjected to in-solution tryptic digestion (Carrera et al., 2013). A total of 100 μg of each bacterial protein extract was thus dried in a speedvac (CentriVap, Labconco Co., USA) and denatured with 25 μL of 8 M urea in 25 mM ammonium bicarbonate at pH 8.0. After 5 min of sonication, DTT was added to a final concentration of 10 mM, and the solution was incubated at 37°C for 1 h. Iodoacetamide was then added to a final concentration of 50 mM, and the solution was incubated for 1 h at room temperature in darkness. Finally, the sample was diluted four times with 25 mM ammonium bicarbonate, pH 8.0, and subjected to enzymatic digestion with trypsin (1:100 protease-to-protein ratio) (Promega, WI, US) at 37°C overnight.

### Shotgun LC-MS/MS analysis

Tryptic digests were acidified with formic acid (FA), cleaned on a C_18_ MicroSpin™ column (The Nest Group, South-borough, MA) and analyzed by LC-MS/MS using a Proxeon EASY-nLC II Nanoflow system (Thermo Fisher Scientific, San Jose, CA) coupled to a LTQ-Orbitrap XL mass spectrometer (Thermo Fisher Scientific) (Carrera et al., 2013, 2016). Peptide separation (2 μg) was performed on a reverse-phase (RP) column (EASY-Spray column, 50 cm × 75 μm ID, PepMap C18, 2 μm particles, 100 Å pore size, Thermo Fisher Scientific) with a 10 mm precolumn (Accucore XL C18, Thermo Fisher Scientific), using a linear 120 min gradient from 5 to 35% solvent B (solvent A: 98% water, 2% ACN, 0.1% FA; solvent B: 98% ACN, 2% water, 0.1% FA) at a flow rate of 300 nL/min. For ionization, a spray voltage of 1.95 kV and a 230°C capillary temperature were used. Peptides were analyzed in positive mode from 400 to 1,600 amu (1 μscan), followed by 10 data-dependent collision-induced dissociation (CID) MS/MS scans (1 μscan) using an isolation width of 3 amu and a normalized collision energy of 35%. Fragmented masses were set in dynamic exclusion for 30 s after the second fragmentation event, and unassigned charged ions were excluded from MS/MS analysis.

### LC-MS/MS mass spectrometry data processing

The results from the LC-MS/MS spectra were searched against the *S. aureus* UniProt/TrEMBL database (154,602 protein sequence entries) using SEQUEST-HT (Proteome Discoverer 2.1, Thermo Fisher Scientific). The following constraints were used: semi-tryptic cleavage with up to two missed cleavage sites and tolerance windows of 1.2 Da for precursor ions and 0.6 Da for MS/MS fragment ions. The variable modifications that were allowed were as follows: methionine oxidation (+15.99 Da), carbamidomethylation of Cys (+57.02 Da) and acetylation of the N-terminus of the protein (+42.0106 Da). The database search results were subjected to statistical analysis with the Percolator algorithm (Kall et al., 2007). To validate the peptide assignments, the false discovery rate (FDR) was kept below 1%.

### Functional pathways and gene ontology (GO) analysis

The final list of non-redundant protein IDs (column “Gene name” in Supplemental Data 1) was submitted to the PANTHER program (http://www.pantherdb.org/) for classification based on two main types of annotations: protein class and biological process. The whole *S. aureus* genome was selected as a reference set. The statistical significance of representation for the analysis was also provided. Functional pathways were also obtained using PANTHER software. For this methodology, all of the orthologous *S. aureus* gene ID entries were used as a reference set. The pathway analysis data were clustered, providing an estimation of the statistical significance of over- or underrepresentation based on the GO terms of the proteins in the proteome.

### Euclidean hierarchical clustering

Euclidean hierarchical clustering analysis of the data was performed using the function heatmap.2 of the statistical package R version (v) 3.0.1 (http://www.r-project.org). The Ggplots v.3.0.1 package, the Euclidean distance metric and the complete linkage for the agglomeration method were used as parameters.

### Network analysis

Network analysis was performed by submitting the orthologous *S. aureus* gene IDs to STRING software v.10.0 (http://string-db.org/) (Szklarczyk et al., 2015). STRING is a large database of known and predicted protein interactions. Proteins were represented with nodes, and interactions were represented with continuous lines. All the edges were supported by at least one reference from the literature or from canonical information stored in the STRING dataset. The confidence score was fixed at ≥0.7 (high confidence). Cluster networks were created using the MCL algorithm that is at the STRING website, and a default value of 2 was selected for all analyses.

### Virulence factors and antibiotic resistance proteins

Virulence factors were identified using the Virulence Factors of Pathogenic Bacteria Database (VFDB) (http://www.mgc.ac.cn/VFs/), and antibiotic resistance proteins were identified using the Comprehensive Antibiotic Resistance Database (https://card.mcmaster.ca/). In addition, we extended the analysis to include virulence factors that are registered in several scientific publications.

### Selection of potential peptide biomarkers

To study specificity, we used BLASTp algorithm on each peptide identified by LC-MS/MS to determine homologies and exclusiveness with protein sequences registered in the NCBI database (Altschul et al., 1990).

## Results and discussion

### Strategy for the shotgun and functional proteomics analysis of foodborne strains of *Staphylococcus aureus*

**Figure 1:**
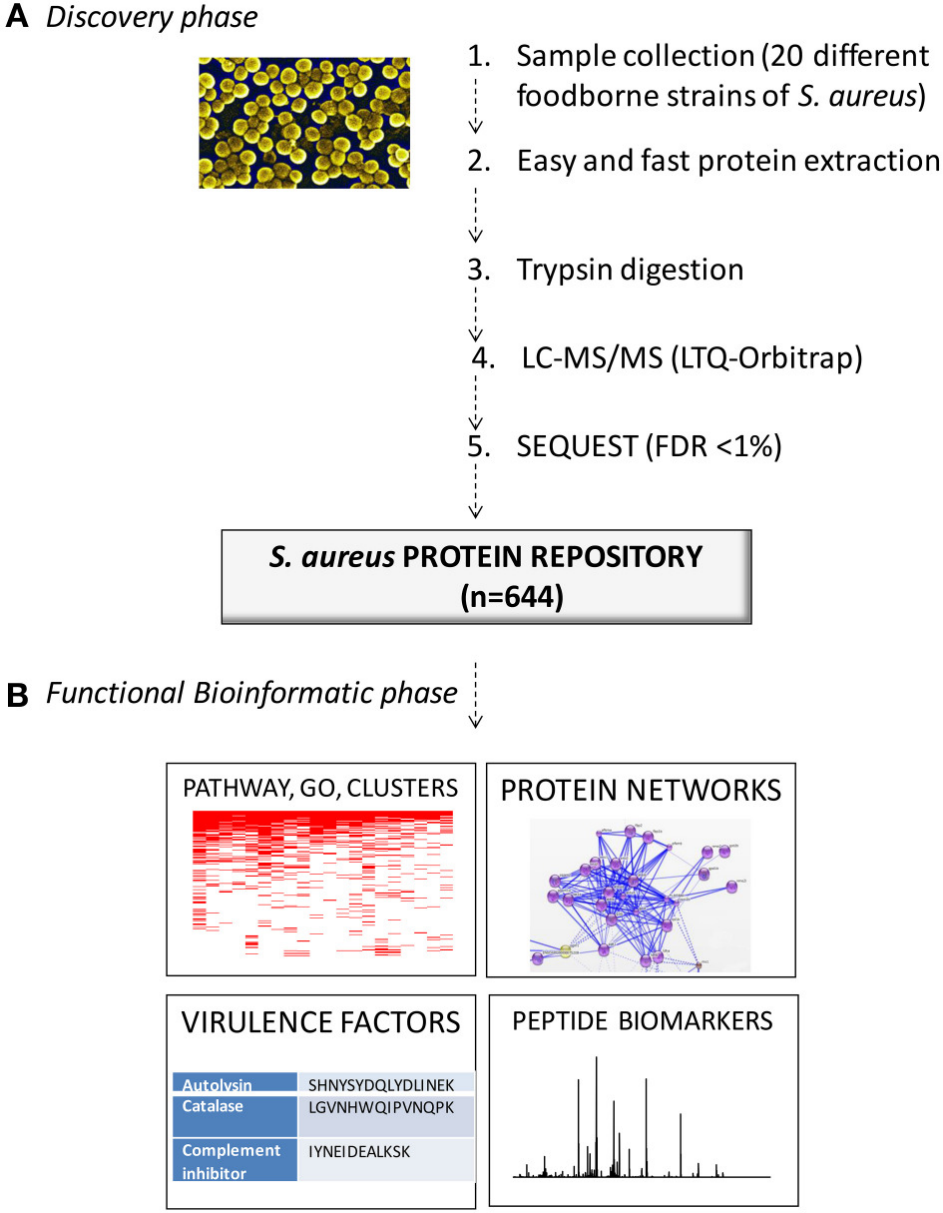
Workflow of the present study. **(A)** Discovery phase: a shotgun proteomics approach (protein extraction, trypsin digestion, LC-MS/MS analysis and database searching) was used to generate a reference protein dataset for the 20 different foodborne strains of *S. aureus*. **(B)** Functional bioinformatics phase: this reference dataset was subjected to analyses of (i) functional pathways, GO, and hierarchical clustering, (ii) functional networks, (iii) virulence factors and (iv) the selection of potential species-specific peptide biomarkers.

The strategy used in this study is illustrated in Figure 1. This study integrates two consecutive steps: (a) the Discovery Phase and (b) the Functional Bioinformatics Phase.

In the first stage (Discovery Phase), a shotgun proteomics approach was used to generate a reference protein dataset for the 20 different foodborne strains of *S. aureus* (Böhme et al., 2012). Shotgun proteomics refers to the analysis of a mixture of proteins that are digested with a protease, and the resulting mixture of peptides are then analyzed by LC-MS/MS. The spectra obtained are assigned to peptide sequences using database-searching algorithms, and the identification of these peptides then allows the identification of the proteins present in the complex mixture.

In the second stage (Functional Bioinformatics Phase), the reference protein dataset was subjected to several *in-silico* bioinformatics analyses such as (i) functional pathways, GO and hierarchical clustering analysis, (ii) functional networks analysis, (iii) finding virulence factors and (iv) the selection of potential species-specific peptide biomarkers. This functional bioinformatics phase is not a new approach, but this study is the first time that it has been applied to the analysis of 20 different foodborne strains of *S. aureus*.

The detailed results of each stage are shown in the following sections.

### Discovery phase

#### Reference protein dataset for foodborne strains of *Staphylococcus aureus*

A total of 1135 non-redundant peptides, corresponding to 644 different annotated non-redundant proteins, compose the present reference dataset for foodborne strains of the pathogenic bacteria *S. aureus* (see complete non-redundant dataset Excel page in Supplemental Data 1). This dataset was obtained following a shotgun proteomics approach for 20 different *S. aureus* strains. This final compilation was created by merging results from a total of 10,945 spectra. Although some intracellular proteins are present, due to the ease of the protein preparation procedure the majority of the identified proteins corresponded to surface-associated proteins (see the cellular location column in Supplemental Data 1). The complete lists of peptides and proteins for each of the 20 different *S. aureus* strains are available in Supplemental Data 1.

In addition, we compared the present dataset with a previous publication that includes the most comprehensive *S. aureus* HG001 protein map (2088 proteins) (Depke et al., 2015). *S. aureus* HG001 is a model strain for the analysis of *S. aureus* in host-pathogen interaction studies. We identified an overlap of 228 proteins and 393 non-redundant peptides between both datasets (Supplemental Data 2).

To the best of our knowledge, the present dataset is the largest dataset of peptides and proteins for foodborne *S. aureus* strains identified to date. This valuable protein repository will add new and significant contents to universal public protein databases and will be very useful for further investigations.

In order to gather a greater number of functional insights, the present repository was next investigated by performing several functional *in-silico* analyses, including (i) functional pathways, GO enrichment and hierarchical clustering analysis, (ii) functional networks analysis, (iii) finding virulence factors and (iv) the screening of potential species-specific peptide biomarkers.

### Functional bioinformatics phase

#### Functional pathways, gene ontology (GO) and hierarchical clustering

**Figure 2:**
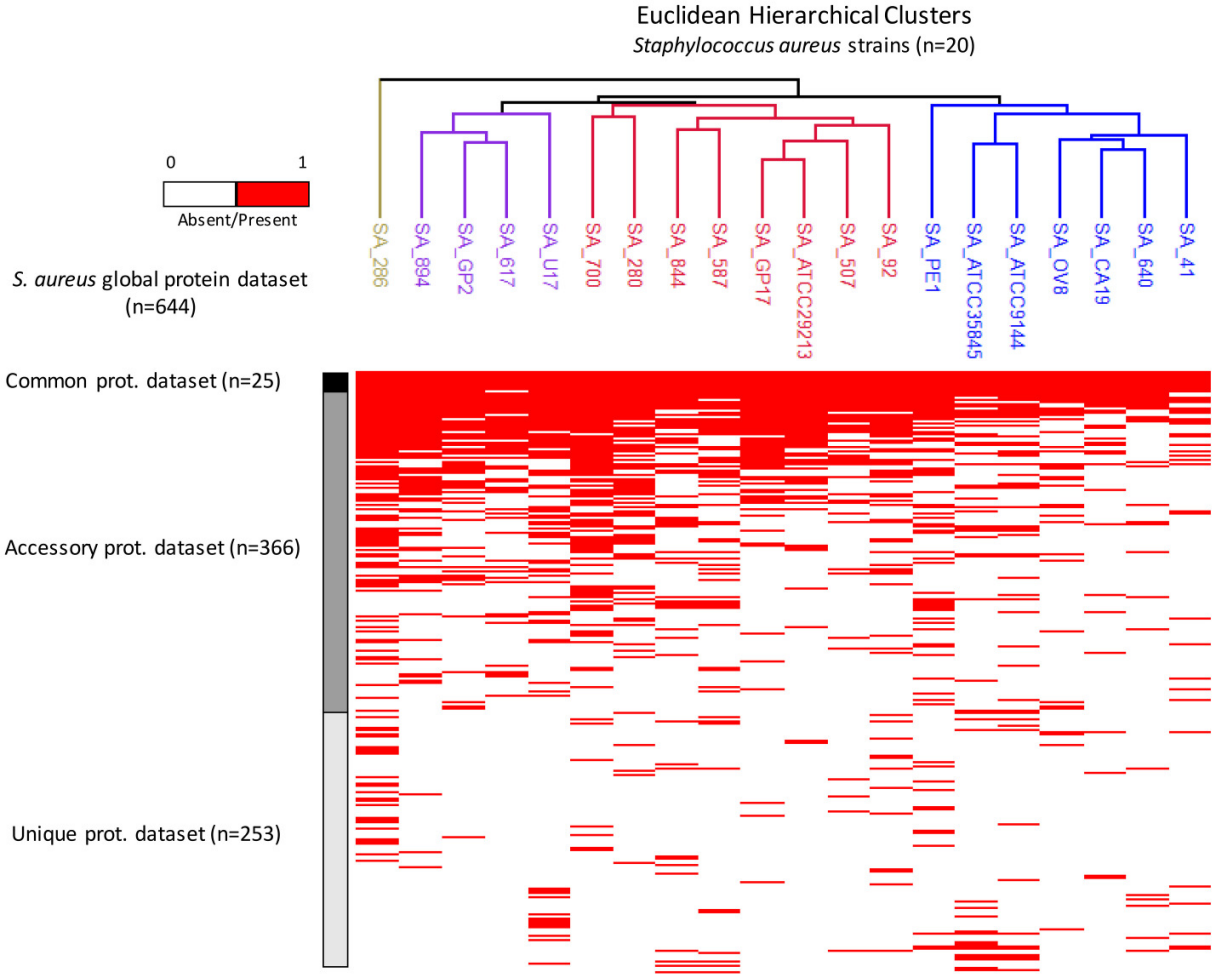
Heat map from the shotgun proteomics analysis of 20 foodborne strains of *S. aureus*. Every red bar corresponds to the presence or absence of a particular protein. Euclidean hierarchical distances were sorted for all *S. aureus* strains. Four main clusters can be differentiated: (i) a global protein repository (*n* = 644 different proteins); (ii) a common protein repository (*n* = 25 proteins); (iii) an accessory protein repository (≥2 strains but not in all strains) (*n* = 366 proteins); and (iv) a unique protein repository (*n* = 253 proteins).

Analysis of the 20 closely related foodborne strains of *S. aureus* is represented by the heat map of Figure 2. Every red bar corresponds to the presence or absence of a particular protein (Supplemental Data 3). Four main clusters were differentiated: (i) the reference global protein repository, containing all the proteins identified in the study (*n* = 644 different proteins); (ii) the common protein repository, whose proteins are represented in all bacterial strains (*n* = 25 proteins); (iii) the accessory protein repository, whose proteins are represented in some (≥2 strains) but not all strains (*n* = 366 proteins); and (iv) the unique protein repository, whose proteins are represented in single strains (*n* = 253 proteins) (Figure 2).

The four types of protein repositories (global, common, accessory and unique) of the foodborne strains of *S. aureus* were individually investigated using functional bioinformatics tools, such as functional pathways analysis and GO-term enrichment. PANTHER analysis revealed the presence of 15 different protein classes in the complete global protein repository (Table S1). The most prominent classes were the nucleic acid-binding proteins (45.10%), transferases (10.4%), oxidoreductases (10.3%) and hydrolases (8.7%). Nucleic acid-binding proteins were also the most prominent protein class in all other types of protein repositories (common, accessory and unique) (Table S2). Nucleic acid-binding proteins, transferases, oxidoreductases and hydrolases are also involved in the virulence mechanism of *S. aureus* (Altschul et al., 1990; Kaito et al., 2006; Bonar et al., 2015; Depke et al., 2015). Results from the evaluation of biological functions by PANTHER revealed the 10 most represented biological functions (Table S1). Among them, metabolic processes are the most relevant biological processes, representing 46.8% of the global, 52.2% of the common, 45.7% of the accessory and 48.8% of the unique protein repository (Tables S1, S2).

Functional pathways were further investigated by PANTHER, and 15 significant canonical pathways were identified in the complete global protein repository (Table S3). The top scored pathways were also identified in the global, common, accessory and unique repositories and included proteins involved in (a) energy metabolism (glycolysis), (b) anabolism (purine and pyrimidine metabolism), and (c) peptidoglycan biosynthesis (the major constituent of the bacterial cell wall) (Tables S3, S4; Crosby et al., 2016).

Finally, all *S. aureus* strains were sorted by Euclidean hierarchical distance (Figure 2). Three main clusters were differentiated, and they corresponded to the production of different enterotoxin types. Enterotoxins A, B, C, D, and E are among the most common staphylococcal toxins associated with food poisoning worldwide. Information about enterotoxin production has been previously obtained by collaborators using multiplex PCR and the same strains (Morandi et al., 2007; Giebel et al., 2010). The cluster corresponding to the SA_92, SA_507, SA_ATCC29213, SA_GP17, SA_587, SA_844, SA_280, and SA_700 strains was grouped together by its members' mutual production of enterotoxin D. The cluster that included the SA_41, SA_640, SA_CA19, SA_OV8, SA_ATCC9144, SA_ATCC35845, and SA_PE1 strains was grouped together by its members' potential production of enterotoxin A. Finally, the cluster including the SA_U17, SA_617, SA_GP2, and SA_894 strains was grouped together by its members' potential production of enterotoxin C. These results are in accordance with previous results obtained by our group using MALDI-TOF mass fingerprinting (Böhme et al., 2012).

#### Network analysis

**Figure 3:**
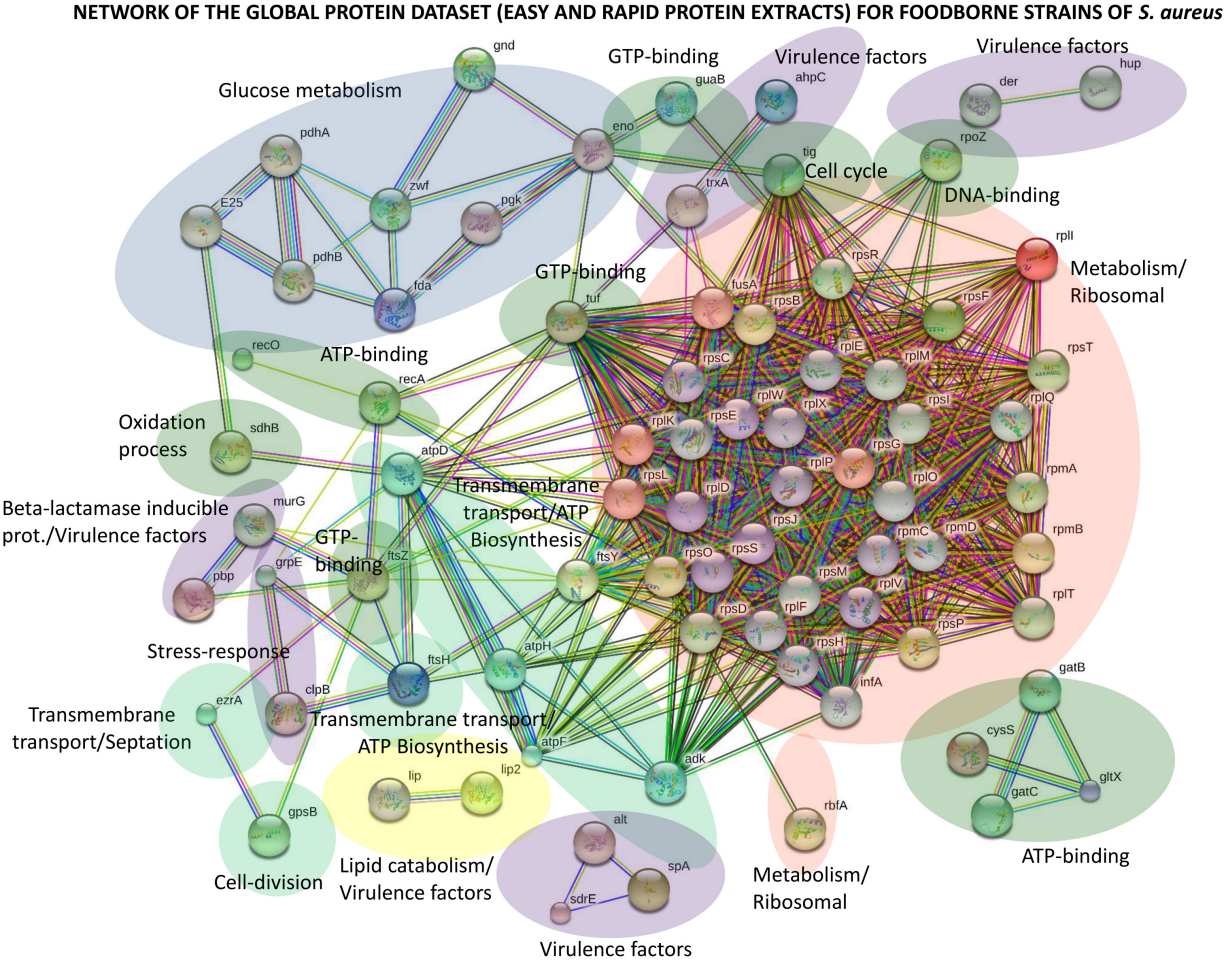
Protein interactome network for the global protein repository of foodborne strains of *S. aureus* using STRING v.10.0 software. High-confidence interactions (≥0.7) in STRING software were selected for this study. The final network for the global protein repository is composed of 77 nodes (proteins) and 769 edges (interactions).

STRING v.10.0 software (Szklarczyk et al., 2015) was used to individually investigate the four types of protein repositories of *S. aureus* strains (global, common, accessory and unique) by protein networks (Figure 3 and Figures S1, S2). Every protein-protein interaction was assigned to a network according to its confidence score. To minimize false positives as well as false negatives, all predicted interactions tagged as “high-confidence” (≥0.7) in STRING software were selected for this study. The final network for the global protein repository was thus composed of 77 nodes (proteins) and 769 edges (interactions) (Figure 3). Of all the interactions, 600 edges were labeled “highest-confidence” (≥0.9). This protein network represents the first comprehensive interactomics map for foodborne strains of *S. aureus*.

Topological analysis of this predicted global protein network demonstrates a centralized interplay, with highly connected nodes and several sparsely connected subnetworks (Figure 3). The main central subnetwork is composed of 33 nodes and referred to as the metabolic, ribosomal and nucleic acid-binding network (Figure 3). The results describing this subnetwork agree with the top biological processes obtained using PANTHER (Table S1). Other relevant subnetworks are composed of nodes involved in glucose metabolism, lipid catabolism and ATP biosynthesis (Figure 3). These nodes are attributed to metabolic linkages whose reactions involve proteins that share common metabolites. It is important to emphasize that several nodes are attributed to virulence factors of the bacterium (Figure 3). Thus, beta-lactam-inducible proteins such as autolysins, thioredoxins and lipases were identified in this network.

In the common protein dataset, the relevant network map is attributed essentially to metabolic/ribosomal processes (Figure S1). All these proteins are involved in functions that are essential for growth, survival and contributions to the virulence of the bacterium. The proteins identified in the accessory and unique protein repositories are involved in glucose metabolism, metabolic/ribosomal processes, virulence and lipid catabolism (Figures S1, S2).

With respect to extracellular proteins, strains SA_ATCC9144 and SA_ATCC35845, considered non-enterotoxigenic (Kall et al., 2007; Morandi et al., 2007), present the extracellular fibrinogen-binding protein Efb. Efb is an extracellular protein that binds specifically to components of human plasma (fibrinogen and platelets) and plays an immunosuppressive role by interfering with the complement system. Efb is thus an essential protein for *S. aureus* virulence that does not produce enterotoxins (Posner et al., 2016).

Compared with the previous study performed on the host-pathogen interaction of *S. aureus* strain HG001 (Depke et al., 2015), the results of the overlapping proteins (228 proteins) showed a common main network composed of metabolic, ribosomal and nucleic acid-binding proteins (Figure S3). Additionally, several nodes are attributed to virulence factors of the bacterium such as autolysins, immunoglobulin-binding proteins (spa and sarA), thioredoxins and lipases, which control the pathogenesis and biofilm formation of the bacteria. We also found the HTH-type transcriptional regulator protein mgrA in the common dataset; this protein is a regulatory protein involved in autolytic activity, virulence and multidrug resistance. These results demonstrate that this protein interacts with surface protein A (spa), leading to its repression and thus causing clumping, which is a mechanism used by pathogens that resist clearance by the immune system (Crosby et al., 2016). In addition, we found the alkyl hydroperoxidase protein AhpC in the common dataset. This protein reduces alkyl hydroperoxides more effectively than H_2_O_2_, is a critical element of the antioxidant defense system of bacteria and may be a suitable target for the development of novel anti-microbial pathogenic strategies (Cosgrove et al., 2007).

Further study of these networks will be very useful for the development of new therapeutic treatments for *S. aureus* food intoxications.

#### Finding virulence factors

Virulence factors were identified using the Virulence Factors of Pathogenic Bacteria Database (VFDB), which includes 1,796 virulence factors (as of 1 Nov 2017) (http://www.mgc.ac.cn/VFs/). In addition, we extended the analysis to include virulence factors that have been registered in scientific publications (Zecconi and Scali, 2013).

Table 2 summarizes the list of 102 peptides, belonging to 27 virulence factors, identified in the foodborne strains of *S. aureus*. Five main functional properties of these peptides were characterized: adherence, antibiotic resistance, immune evasion, invasion and phage lysis.

**Table 2:** Virulence factors peptides identified for foodborne strains of *S. aureus*.

**Function**	**Protein**	**Peptide**	**100% homology with protein NCBI database**
Adherence	ABC transporter, family 5	K.AEEMFLGDAPVAPIYQK.G	*S. aureus, S. schweitzeri, S. haemolyticus*
	ABC transporter	K.QHHLFNPPAYDDFVEPSLYK.G	*S. aureus, S. schweitzeri, S. haemolyticus*
	Manganese ABC transporter substrate-binding protein	K.QYGITPGYIWEINTEK.Q	*S. aureus, S. schweitzeri, S. haemolyticus, S. argenteus, S. carnosus, S. condimenti*
	Nickel ABC transporter substrate-binding protein	K.SIAPYETDVPVK.I	*S. aureus, S. schweitzeri, S. haemolyticus, S. simiae*
	Nickel ABC transporter substrate-binding protein	K.VTPEGIYLIDYR.T	*S. aureus*
	Nickel ABC transporter substrate-binding protein	R.DVPNSYIAYNDQIVAANSK.V	*S. aureus*
	Clumping factor	V.GTLIGFGLLSSK.E	*S. aureus*
	Elastin-binding protein (EbpS)	K.EAENNTEHSTVSDKSEAEQSQQPKPYF.A	*S. aureus*
	Elastin-binding protein (EbpS)	R.IAIQYYGSGSPENVEK.I	*S. aureus, S. haemolyticus, S. schweitzeri, S. simiae, S. cohnii, S. sciuri, S. microti, S. equorum*
	Elastin-binding protein (EbpS)	K.DAENNTEYSTVSDKSEAEQSQQPKPYF.A	*S. aureus*
	Extracellular adherence protein Eap	K.DSQLSYLDLGNKVK.A	*S. aureus, S. schweitzeri, S. haemolyticus, E. coli*
	Serine-aspartate repeat protein E	F.AVAQPAAVASNNVNDLIK.V	*S. aureus*
Adherence/Invasion	Phenol-soluble modulin	-.MTGLAEAIANTVQAAQQHDSVK.L	*S. aureus, S. argenteus*
	Phenol-soluble modulin	-.MGIIAGIIK.V	*S. aureus*
	Phenol-soluble modulin	V.IKSLIEQFTGK.-	*S. aureus*
	Phenol-soluble modulin	K.VIKSLIEQFTGK.-	*S. aureus*
	Phenol-soluble modulin	-.MGIIAGIIKVIK.S	*S. aureus*
	Phenol-soluble modulin	K.GLIEKFTGK.-	*S. aureus*
	Phenol-soluble modulin	K.FIKGLIEKFTGK.-	*S. aureus*
	Phenol-soluble modulin	G.IIAGIIKFIK.G	*S. aureus*
	Phenol-soluble modulin	G.IIKFIKGLIEK.F	*S. aureus*
	Phenol-soluble modulin	K.IIKAIIDIFAK.-	*S. aureus*
	Phenol-soluble modulin	K.AIIDIFAK.-	*S. aureus*
	Phenol-soluble modulin	I.IKAIIDIFAK.-	*S. aureus*
	Phenol-soluble modulin	-.MAIVGTIIK.I	*S. aureus*
	Thioredoxin	K.SPEQVESFLAETFK.-	*S. aureus, S. argenteus, S. gallinarum, S. haemolyticus, S. schweitzeri, S. xylosus*
Antibiotic resistance	Beta-lactamase protein	R.YEIELNYYSPK.S	*S. aureus, S. epidermis, S. haemolyticus, S. warneri, S. argenteus*
	Beta-lactamase protein	K.YNANIGVYALDTK.S	*S. aureus, S. epidermis, S. haemolyticus, S. argenteus, S. intermedius*
	Beta-lactamase protein	R.NDVAFVYPK.N	*S. aureus, S. epidermis, S. haemolyticus, S. argenteus, S. intermedius, S. hominis*
	Beta-lactamase protein	K.VADKSGQAITYASRNDVAFVYPK.N	*S. aureus, S. epidermis, S. haemolyticus, S. argenteus, S. intermedius, S. hominis*
	Beta-lactamase protein	K.AINSAILLEQVPYNKLNKK.I	*S. aureus, S. pneumonia, S. epidermis, S. haemolyticus, S. capitis, S. warneri*
	Beta-lactamase protein	K.KVHINKDDIVAYSPILEK.Y	*S. aureus, S. saprophyticus, S. equorum, S. epidermis, S. haemolyticus, S. warneri, S. argenteus*
	Beta-lactamase protein	G.DKVTNPVRYEIELNYYSPK.S	*S. aureus, S. epidermis, S. haemolyticus, S. equorum, S. capitis, S. warneri*
	Beta-lactamase protein	K.VHINKDDIVAYSPILEK.Y	*S. aureus, S. saprophyticus, S. equorum, S. epidermis, S. haemolyticus, S. warneri, S. argent*.
	Beta-lactamase protein	R.NDVAFVYPK.N	*S. aureus, S. epidermis, S. haemolyticus, S. argenteus, S. intermedius*
	Beta-lactamase protein	K.SGQAITYASRNDVAFVYPK.G	*S. aureus, S. epidermis, S. sciuri, S. haemolyticus, S. saprophyticus, S. equorum, S. capitis*
	Beta-lactamase protein	R.NDVAFVYPK.G	*S. aureus, S. sciuri, S. saprophyticus, S. epidermis, S. haemolyticus, S. capitis, S. warneri*
	Beta-lactamase protein	K.AINSAILLEQVPYNKLNK.K	*S. aureus, S. epidermis, S. haemolyticus, S. saprophyticus, S. equorum, S. capitis, S. warneri*,
	Beta-lactamase protein	K.AINSAILLEQVPYNK.L	*S. aureus, S. epidermis, S. haemolyticus, S. saprophyticus, S. equorum, S. capitis, S. warneri*,
	Beta-lactamase protein	E.LGDKVTNPVRYEIELNYYSPK.S	*S. aureus, S. epidermis, S. saprophyticus, S. haemolyticus, S. equorum, S. argenteus, S. capitis*
	Beta-lactamase protein	K.YSDNTANNKIINEIGGIKK.I	*S. aureus, S. epidermis, S. haemolyticus, S. warneri, S. interemedius*
	Beta-lactamase protein	K.AINSAILLEQVPYNKLNKK.V	*S. aureus, S. saprophyticus, S. equorum, S. epidermis, S. haemolyticus, S. warneri*
	Beta-lactamase protein	K.KYNAHIGVYALDTK.S	*S. aureus, S. epidermis, S. haemolyticus, S. saprophyticus, S. equorum, S. capitis, S. warneri*
	Transcriptional regulator, MarR family	K.LISELEEYIK.N	*S. aureus*
	Transcriptional regulator, MarR family	K.LVKLDKPNLNIDQR.L	*S. aureus, S. haemolyticus, S. argenteus*
Immune evasion	Catalase	R.LGVNHWQIPVNQPK.G	*Staphylococcus* spp., *Streptococcus pneumoniae, Streptococcus equi* subsp. *equi*
	Complement inhibitor	K.IYNEIDEALKSK.Y	*S. aureus, Staphylococcus phage 13, Staphylococcusphage phiNM3*
	Extracellular fibrinogen-binding protein (Efb)	K.AVNLVSFEYK.V	*S. aureus, S. schweitzeri*
	Extracellular fibrinogen-binding protein (Efb)	K.LIQAQNLVR.E	*S. aureus, S. schweitzeri*
	Extracellular fibrinogen-binding protein (Efb)	K.VVDAQKAVNLFKR.T	*S. aureu S. argenteus, S. haemolyticus, S. schweitzeri*.
	Extracellular fibrinogen-binding protein (Efb)	R.AVNLIHFQHSYEK.K	*S. aureus, S. argenteus, S. haemolyticus*.
	LukF-PV	K.SYDKDTLVLK.A	*S. aureus, S. pseudintermedius, S. intermedius*,
	Peroxiredoxin	K.EILPFTAQAFD.-	*S. aureus, S. argenteus, S. haemolyticus*
	Peroxiredoxin	K.TLQPGLDLVGK.I	*S. aureus, S. argenteus, S. haemolyticus*
	Peroxiredoxin	R.GTFIIDPDGVVQASEINADGIGR.D	*S. aureus, S. argenteus, S. haemolyticus*
Invasion	Cysteine protease peptide/Staphopain	K.TESIPTGNNVTQLK.Q	*S. aureus, S. schweitzeri, E. coli*
	Cysteine protease peptide/Staphopain	K.YTINVSSFLSK.V	*S. aureus*
	Cysteine protease peptide/Staphopain	K.YTINVSSFLSK.A	*S. aureus*
	Cysteine protease peptide/Staphopain	R.FLHPNLQGQQFQFTGLTPR.E	*S. aureus, S. haemolyticus*
	Cysteine protease peptide/Staphopain	R.MTTYNEVDNLTK.N	*S. aureus, S. schweitzeri, E. coli*
	Gram-posit. signal peptide protein, YSIRK family	R.NGFIQSLKDDPSQSTNVLGEAK.K	*S. aureus, S. schweitzeri*
	Gram-posit. signal peptide protein, YSIRK family	R.TLPQRQQTSR.R	*S. aureus*
	Lipase	K.AYEASISAFGSNYDR.A	*S. aureus, S. argenteus, S. haemolyticus, S. schweitzeri*
	Lipase	K.HGGEISPLFK.G	*S. aureus, S. haemolyticus*
	Lipase	K.QGYNVHQASVSAFGSNYDR.A	*S. aureus, S. schweitzeri, S. simulans, S. xylosus*
	Lipase	R.VDFGLAQWGLK.Q	*S. aureus, S. argenteus, S. caprae, S. haemolyticus, S. schweitzeri*.
	Malate:quinone reductase	R.TLLFGPFANVGPK.F	*S. aureus, Staphylococcus spp*.
	Peptidase	R.AFGLIDEDQIVGK.V	*S. aureus, S. argenteus, S. haemolyticus, S. schweitzeri, S. schleiferi*
	Tautomerase	K.NLVSEVTDAVEK.T	*S. aureus, S. haemolyticus*
	Tautomerase	R.QAIHVVIEEMKPNHYGVAGVR.K	*S. aureus, S. haemolyticus*
	Universal stress family UpsA	K.ALEDYGIDYDQIIVR.G	*S. aureus, S. capitis, S. argenteus, S. schweitzeri, S. haemolyticus*
	Universal stress family UpsA	R.FIVGSVSESIVR.H	*S. aureus, S. argenteus, S. schweitzeri*
	Universal stress family UpsA	R.TEELPADFQPQVATTQLR.E	*S. aureus, S. simiae, S. schweitzeri*
	Virulence factor EsxA	K.SQSYGQGSDQIRQILSDLTR.A	*S. aureus, S. argenteus*
	Virulence factor EsxA	R.AQGEIAANWEGQAFSR.F	*S. aureus, S. argenteus, S. lugdunensis, S. epidermidis*
	Virulence factor EsxA	R.FEEQFQQLSPK.V	*S. aureus, S. argenteus, S. lugdunensis, S. epidermidis*
Phage lysis	N-Acetylmuramoyl-L-alanine amidase	K.IMSLNGLNNFFIYPGQK.L	*S. aureus, S. argenteus, S. schweitzeri, S. haemolyticus, S. simiae, S. simulans*
	N-Acetylmuramoyl-L-alanine amidase	K.TIDDSTSDSNNIIDFIYK.N	*S. aureus*
	N-Acetylmuramoyl-L-alanine amidase	R.LNGLNNFFIYPGQK.L	*S. aureus, S. argenteus, S. schweitzeri, S. haemolyticus, S. hominis, S. lugdunensis, S. simiae, S. simulans*
	Autolysin	R.IIETAPTDYLSWGVGAVGNPR.F	*S. aureus, S. epidermidis, S. schweitzeri, S. argenteus*,
	Autolysin	K.AEVKNPTQNISGTQVYQDPAIVQPK.A	*S. aureus*
	Autolysin	K.AYLAVPAAPK.K	*S. aureus, S. argenteus*
	Autolysin	K.DLNVQNLGK.E	*S. aureus*
	Autolysin	K.DYNSPTLIGWVK.Q	*S. aureus, S. schweitzeri*
	Autolysin	K.DYNYTYVIK.N	*S. aureus, S. schweitzeri*
	Autolysin	K.EASLGGNKFYLVK.D	*S. aureus, S. argenteus*
	Autolysin	K.EGDVVYNTAKSPVNVNQSYSIK.P	*S. aureus*
	Autolysin	K.FYLVQDYNSGNK.F	*S. aureus, S. schweitzeri, S. argenteus*
	Autolysin	K.GVLENQGAAFNK.A	*S. aureus, S. schweitzeri, S. haemolyticus, S. argenteus*
	Autolysin	K.IGEVGKYFDIPQYK.-	*S. aureus, S. schweitzeri, S. argenteus*
	Autolysin	K.IEEDYTSYFPK.Y	*S. aureus, S. argenteus, S. lugdunensis, S. schweitzeri, S. simiae*
	Autolysin	K.LTVSSLNGVAQINAK.N	*S. aureus*
	Autolysin	K.LYSVPWGTYKQEAGAVSGTGNQTFK.A	*S. aureus*
	Autolysin	K.NQVILTGNNIAQGTFNATK.Q	*S. aureus*
	Autolysin	K.QVAGSVSGSGNQTFK.A	*S. aureus*
	Autolysin	K.SIYLFGTVNGK.S	*S. aureus*
	Autolysin	K.SPVNVMQTYTVKPGTK.L	*S. aureus*
	Autolysin	K.YHNVFGIAAYDNDPLREGIK.Y	*S. aureus*
	Autolysin	K.YKPQVNSSINDYIR.K	*S. aureus*
	Autolysin	R.FINVEIVHTHDYASFAR.S	*S. aureus*
	Autolysin	R.SHNYSYDQLYDLINEK.Y	*S. aureus, S. argenteus, S. carnosus*
	Toxin mazF	K.YKLDKDSVILLEQIR.T	*Staphylococcus* spp.

Regarding the virulence factors implicated in adherence to host cells, the ABC transporter family protein was identified in *S. aureus* (Table 2). This protein is a transport system that plays direct roles in the virulence of bacteria (Lovering et al., 2012). Manganese and nickel ABC transporter substrate-binding proteins were also found in *S. aureus*. These proteins are considered relevant virulence factors that markedly enhance bacterial adherence and induce the invasion of the host cells (Remy et al., 2013). Clumping factor protein was also identified as a virulence factor. This protein is involved in adherence, binds to fibrinogen and is a mediator of *S. aureus*-induced platelet aggregation (Hair et al., 2008). In addition, the extracellular adherence protein Eap was identified as a virulence factor and shows high affinity for fibronectin, fibrinogen and thrombospondin. Eap protein has strong anti-inflammatory properties (Scriba et al., 2008). The elastin-binding protein EbpS was also identified as a virulence factor. This protein is involved in adherence and promotes bacterial colonization (Downer et al., 2002). Serine-aspartate repeat protein E is a cell surface-associated protein that causes human platelet aggregation and is involved in the aggregation of host platelets, thus playing a role in infective endocarditis (O'Brien et al., 2002). Phenol-soluble modulin was also identified as a virulence factor with adherence properties. A total of 13 peptides belonging to phenol-soluble modulin were identified (Table 2). This protein is associated with the enhanced destruction of red and white blood cells, stimulates inflammatory responses and contributes to biofilm development and the dissemination of biofilm-associated infections. Moreover, the pronounced capacity of phenol-soluble modulin to kill human neutrophils after phagocytosis might explain failures in the development of anti-staphylococcal vaccines (Peschel and Otto, 2013). Finally, thioredoxin was identified as a virulence factor with adherence properties, and it is implicated in adhesion to the extracellular matrix and the invasion of epithelial and macrophage-like cells (Lu and Holmgren, 2014).

Virulence factors with antibiotic resistance properties include 16 peptides belonging to the beta-lactamase protein (Table 2). This enzyme is considered a virulence factor with antibiotic resistance properties that provides multiresistance to beta-lactam antibiotics such as penicillins, cephamycins and carbapenems (Lee et al., 2013). In addition, the transcriptional regulator MarR was also identified in *S. aureus*. This protein is a virulence factor that is relevant for being involved in the development of resistance to multiple antibiotics, including tetracycline, penicillins, β-lactams, and fluoroquinolones (Grove, 2013).

The catalase protein, an immune evasion virulence factor, was identified in the present study. Bacteria use this enzyme to combat neutrophils and other phagocytes (Musa et al., 2010). The complement inhibitor protein was also identified as a virulence factor and is an evasion protein that helps bacteria escape attacks from the immune system by blocking C3 convertase (van Wamel et al., 2006). As mentioned earlier, Efb was also identified as a virulence factor, is a potent inhibitor of platelet aggregation and plays relevant immunosuppressive roles (Posner et al., 2016). A Panton-Valentine leukocidin protein, LukF-PV, is also a virulence factor. This cytotoxin creates pores in the membranes of infected cells and causes leukocyte destruction and necrotizing pneumonia (Gillet et al., 2002). Finally, peroxiredoxin was also identified as a virulence factor for macrophages and activated neutrophils (Weber et al., 2004).

The cysteine protease staphopain was identified as an invasion virulence factor (Kantyka et al., 2011). In addition, a gram-positive signal peptide protein from the YSIRK family was identified in this study. This secreted protein is essential for surface protein anchoring and the peptidoglycan envelope (Dedent et al., 2008). A lipase protein was also identified as a virulence factor. This protein contributes to virulence by binding to collagen and enabling bacteria to persist in the fatty secretions of human skin (Hu et al., 2012). The oxidoreductase malate:quinone reductase and peptidase were identified as virulence factors of *S. aureus* as was a tautomerase protein. Tautomerase is part of the oxidative metabolic pathway and considered a virulence factor of *S. aureus* (Kenichi et al., 2014). Universal stress family protein UpsA is a virulence factor that is implicated in resistance to a large number of stressors such as tetracycline exposure and high temperatures (Jenkins et al., 2011). Finally, the virulence factor EsxA was also identified in the present study; this protein is a critical virulence factor for invasive disease episodes (Sundaramoorthy et al., 2008).

N-acetylmuramoyl-L-alanine amidase was identified in the present results and is considered a virulence factor that is phage lytic. This protein plays a role in the adhesion of *S. aureus* to phages and to eukaryotic cells via its cell wall-binding domain (Campbell et al., 1969). A total of 22 peptides belonging to the autolysin protein were found in the foodborne strains of *S. aureus*. Autolysin is an enzyme that hydrolyzes the peptidoglycan matrix and causes the cell to burst from osmotic pressure. It is similar in function to lysozyme and considered a relevant virulence factor necessary for the full pathogenic virulence of *S. aureus* (Liu et al., 2006). The toxin mazF serves to protect cells against bacteriophages and is induced either during nutrient starvation, upon overexpression of a cell cycle regulation protein, during hypoxia and during antibiotic treatment (Schuster and Bertram, 2016).

The identification of these peptide virulence factors might be very useful for the design and development of new drugs that interfere with the virulence of *S. aureus*.

#### Screening of potential species-specific peptide biomarkers

**Figure 4:**
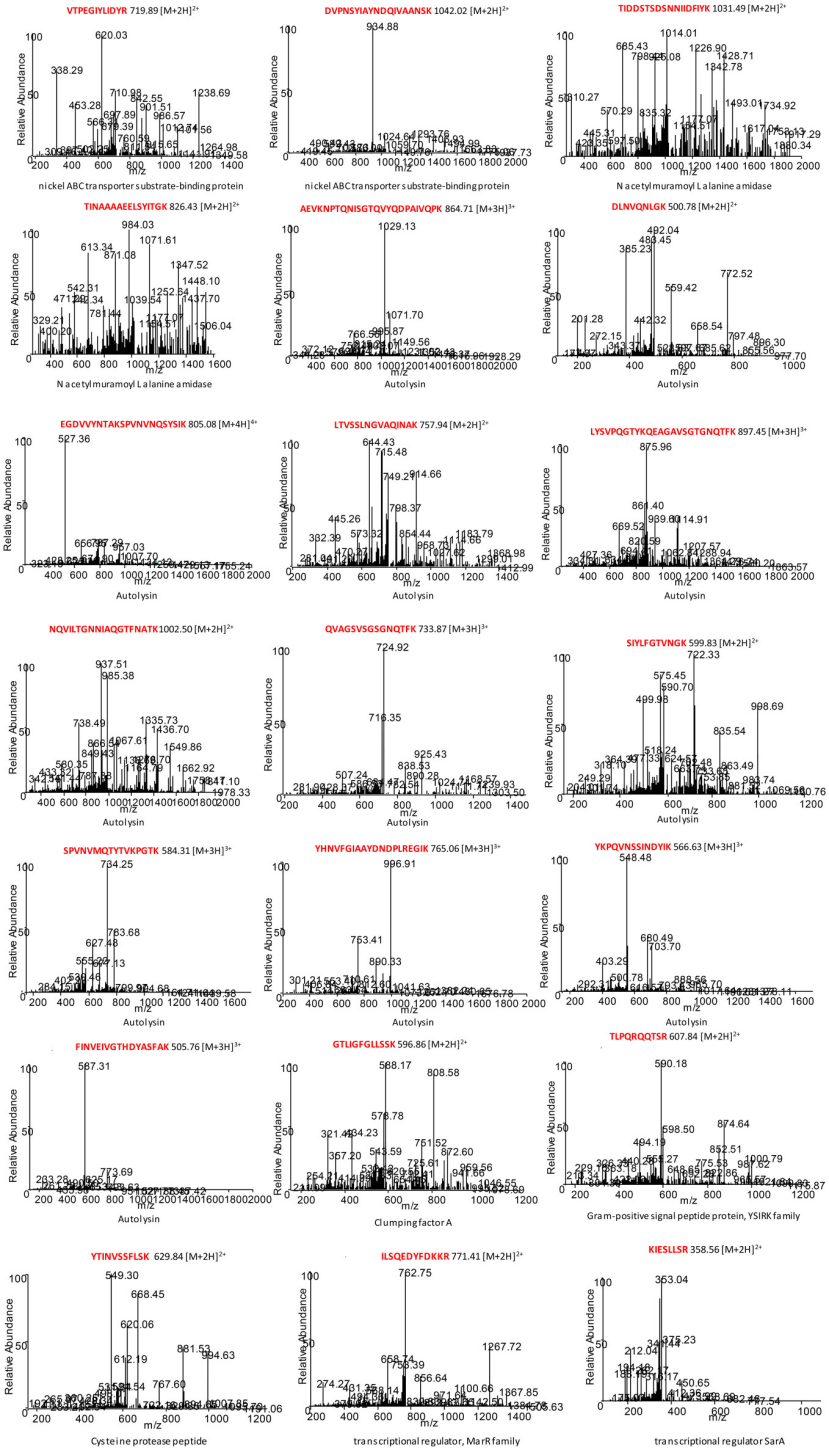
MS/MS spectra for each species-specific peptide biomarker from foodborne *S. aureus*. The corresponding peptides were tested for specificity to *S. aureus* using the BLASTp algorithm.

To define potential peptide biomarkers for foodborne *S. aureus* bacterial identification, we performed an extensive comparison of the proteomics data with respect to the proteins and peptides registered in databases. The appropriate peptides that had been identified by LC-MS/MS were tested for specificity and sequence homology using the BLASTp algorithm (Altschul et al., 1990). Table 3 summarizes the analysis of the 21 species-specific peptide biomarkers proposed for the unequivocal identification of foodborne strains of *S. aureus*. The corresponding spectrum for each of the 21 peptide biomarkers is shown in Figure 4.

**Table 3:** Potential species-specific peptide biomarkers for foodborne *S. aureus*. Specificity found after similarity search using BLASTp.

**Protein**	**Peptide**	**Specific by blastp**
Nickel ABC transporter substrate-binding protein	K.VTPEGIYLIDYR.T	*S. aureus*
Nickel ABC transporter substrate-binding protein	R.DVPNSYIAYNDQIVAANSK.V	*S. aureus*
N-Acetylmuramoyl-L-alanine amidase	K.TIDDSTSDSNNIIDFIYK.N	*S. aureus*
N-Acetylmuramoyl-L-alanine amidase	R.TINAAAAEELSYITGK.-	*S. aureus*
Autolysin	K.AEVKNPTQNISGTQVYQDPAIVQPK.A	*S. aureus*
Autolysin	K.DLNVQNLGK.E	*S. aureus*
Autolysin	K.EGDVVYNTAKSPVNVNQSYSIK.P	*S. aureus*
Autolysin	K.LTVSSLNGVAQINAK.N	*S. aureus*
Autolysin	K.LYSVPWGTYKQEAGAVSGTGNQTFK.A	*S. aureus*
Autolysin	K.NQVILTGNNIAQGTFNATK.Q	*S. aureus*
Autolysin	K.QVAGSVSGSGNQTFK.A	*S. aureus*
Autolysin	K.SIYLFGTVNGK.S	*S. aureus*
Autolysin	K.SPVNVMQTYTVKPGTK.L	*S. aureus*
Autolysin	K.YHNVFGIAAYDNDPLREGIK.Y	*S. aureus*
Autolysin	K.YKPQVNSSINDYIR.K	*S. aureus*
Autolysin	R.FINVEIVHTHDYASFAR.S	*S. aureus*
Clumping factor A	V.GTLIGFGLLSSK.E	*S. aureus*
Gram-positive signal peptide protein, YSIRK family	R.TLPQRQQTSR.R	*S. aureus*
Cysteine protease peptide	K.YTINVSSFLSK.V	*S. aureus*
Transcriptional regulator, MarR family	K.ILSQEDYFDKKR.N	*S. aureus*
Transcriptional regulator Sar-A	K.KIESLLSR.V	*S. aureus*

A total of 21 species-specific peptide biomarkers for foodborne strains of *S. aureus*, belonging to eight different proteins, were selected in this work (Table 3). Among them, we selected two potential species-specific peptide biomarkers (K.VTPEGIYLIDYR.T; R.DVPNSYIAYNDQIVAANSK.V) that belong to the nickel ABC transporter substrate-binding protein and were specific for *S. aureus*. In addition, two peptide biomarkers (K.TIDDSTSDSNNIIDFIYK.N; R.TINAAAAEELSYITG.K) belonging to the N-acetylmuramoyl L-alanine amidase were also chosen. Twelve potential peptide biomarkers belonging to the autolysin protein were chosen as species-specific peptides for foodborne strains of *S. aureus*. Another species-specific peptide biomarker for *S. aureus* (V.GTLIGFGLLSSK.E) belonged to the clumping factor A protein, which is also a virulence factor of *S. aureus* (Hair et al., 2008). The potential peptide biomarker (R.TLPQRQQTSR.R) belongs to the gram-positive signal peptide protein from the YSIRK family and is secreted into the cross wall during peptidoglycan synthesis (Dedent et al., 2008). Another peptide biomarker for foodborne strains of *S. aureus* (K.YTINVSSFLSK.V) belongs to the cysteine protease staphopain, which is a virulence factor that likely contributes to the chronicity of *S. aureus* infections (Kantyka et al., 2011). The potential peptide biomarker (K.ILSQEDYFDKKR.N) belongs to a MarR family transcriptional regulator protein (Lu and Holmgren, 2014). Finally, we selected a potential peptide biomarker (K.KIESLLSR.V) that belongs to the transcriptional regulator SarA, a regulator of virulence determinants (Liu et al., 2006). This protein has recently been considered a novel clinical protein biomarker for *S. aureus* vascular graft infections (Arya et al., 2015).

All of these peptides were proposed here as potential biomarkers for the first time and will be very useful for further investigations using targeted proteomics approaches for the identification of *S. aureus* in real foodstuffs.

## Conclusions

This work presents the first shotgun proteomics study of foodborne strains of *S. aureus*. Using an easy and rapid protein preparation procedure the results allowed the differentiation of several protein datasets (global, common, accessory and unique) that were used to determine relevant functional pathways and to differentiate *S. aureus* strains into different Euclidean hierarchical clusters. Moreover, a predicted protein-protein interaction network for foodborne *S. aureus* was created. Most of the proteins were grouped under pathways and networks related to energy, lipid metabolism and virulence pathways. In addition, 27 different virulence factors were identified. Most of these factors corresponded to autolysins, N-acetylmuramoyl-L-alanine amidases, phenol-soluble modulins, extracellular fibrinogen-binding proteins and virulence factors EsxA. Finally, a screening of 21 potential species-specific peptide biomarkers, belonging to eight different proteins, was performed to find unique potential peptide biomarkers for foodborne *S. aureus*. These results constitute the major dataset of peptides and proteins from foodborne *S. aureus* strains. This repository will be very useful in further studies, for the development of new therapeutic treatments for *S. aureus* food intoxications and for microbial source-tracking in foodstuffs.

## Author contributions

All authors listed have made a substantial, direct and intellectual contribution to the work, and approved it for publication.

### Conflict of interest statement

The authors declare that the research was conducted in the absence of any commercial or financial relationships that could be construed as a potential conflict of interest.
