# Supplementary material for: Characterization of Foodborne Strains of Staphylococcus aureus by Shotgun Proteomics: Functional Networks, Virulence Factors and Species-Specific Peptide Biomarkers
Source: Front Microbiol. 2017 Dec 11;8:2458. doi: 10.3389/fmicb.2017.02458 (PMC5732212; doi:10.3389/fmicb.2017.02458)
Supplement: Supplementary file 4 [file DataSheet1.DOCX]

**SUPPLEMENTAL DATA 4**

**Characterization of Foodborne Strains of *Staphylococcus aureus* by Shotgun Proteomics: Functional Networks, Virulence Factors and Species-Specific Peptide Biomarkers**

Mónica Carrera^1^*, Karola Böhme^2^, José M. Gallardo^1^, Jorge Barros-Velázquez^2^,

Benito Cañas^3^ and Pilar Calo-Mata^2^*

^1^Department of Food Technology, Spanish National Research Council (CSIC), Marine Research Institute (IIM), Vigo, Spain

^2^Department of Analytical Chemistry, Nutrition and Food Science, School of Veterinary Sciences, University of Santiago de Compostela (USC), Lugo, Spain

^3^Department of Analytical Chemistry, Complutense University of Madrid (UCM), Madrid, Spain

***CORRESPONDING AUTHOR:** Dr. Pilar Calo-Mata

Department of Analytical Chemistry, Nutrition and Food Science, School of Veterinary Sciences, University of Santiago de Compostela (USC), Campus Lugo, 27002 Lugo, Spain

e-mail: [p.calo.mata@usc.es](mailto:p.calo.mata@usc.es); Phone: +34647344274, Fax: +34982252195

***CO-CORRESPONDING AUTHOR:** Dr. Mónica Carrera

Department of Food Technology, Spanish National Research Council (CSIC), Marine Research Institute (IIM), Vigo, Spain

e-mail: [mcarrera@iim.csic.es](mailto:mcarrera@iim.csic.es); Phone: +34986231930, Fax: +34986292762

**Table S-1.** Protein classes, biological processes and molecular functions for the global protein dataset (easy and rapid protein extracts) of foodborne strains of *S. aureus* by PANTHER.

**Table S-2.** Protein classes, biological processes and molecular functions of the common, accessory and unique protein datasets by PANTHER.

**Table S-3.** Pathway enrichment analysis for the global protein dataset (easy and rapid protein extracts) of foodborne strains of *S. aureus* by PANTHER.

**Table S-4.** Pathway enrichment analysis of the common, accessory and unique protein repositories by PANTHER.

**Figure S-1.** Protein interactome networks for the (a) common proteome and (b) accessory proteome by STRING.


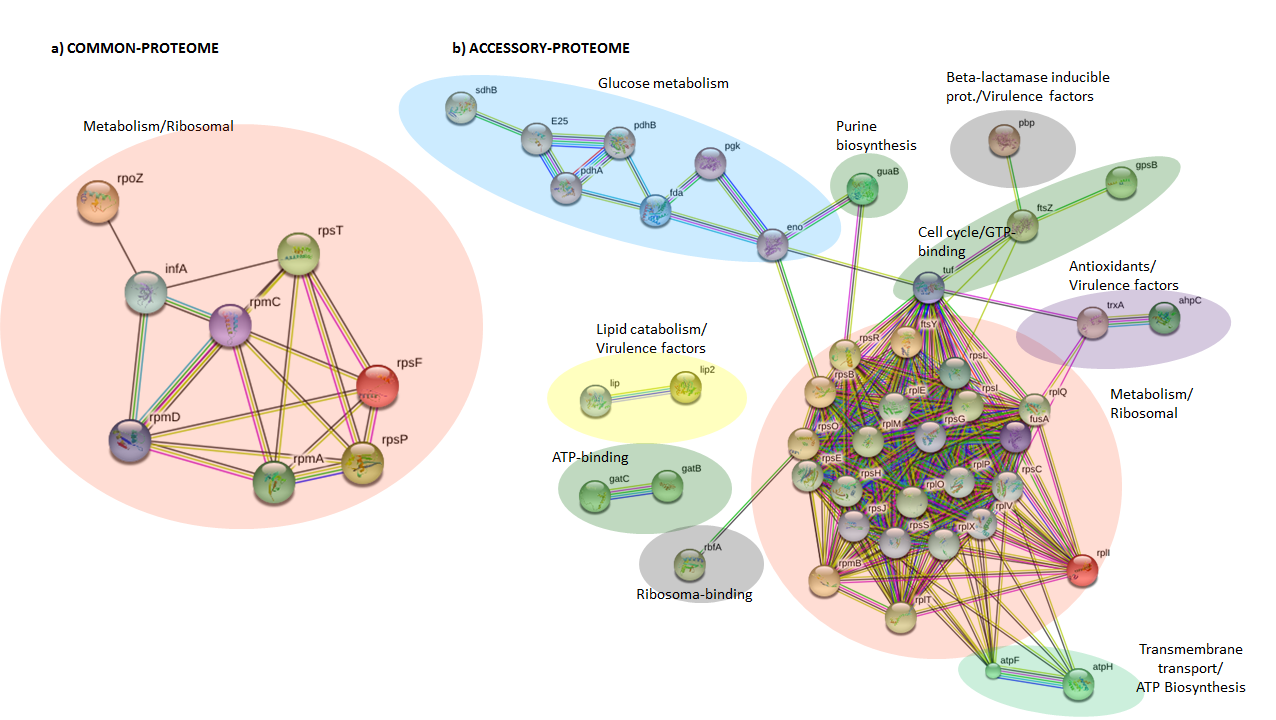


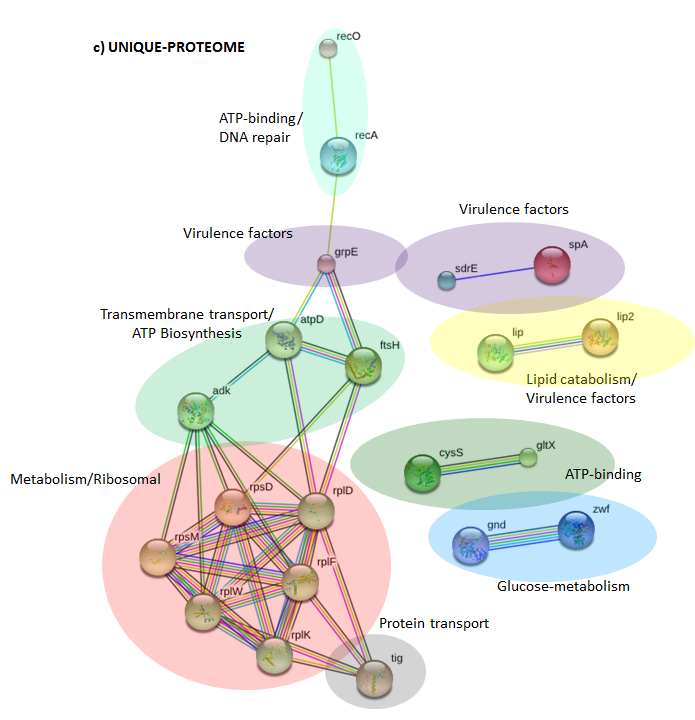
**Figure S-2**. Protein interactome network for the (c) unique proteome by STRING.

**Figure S-3.** Protein interactome network for the common proteins identified in the Depke et al. 2015 article and in the present manuscript by STRING.


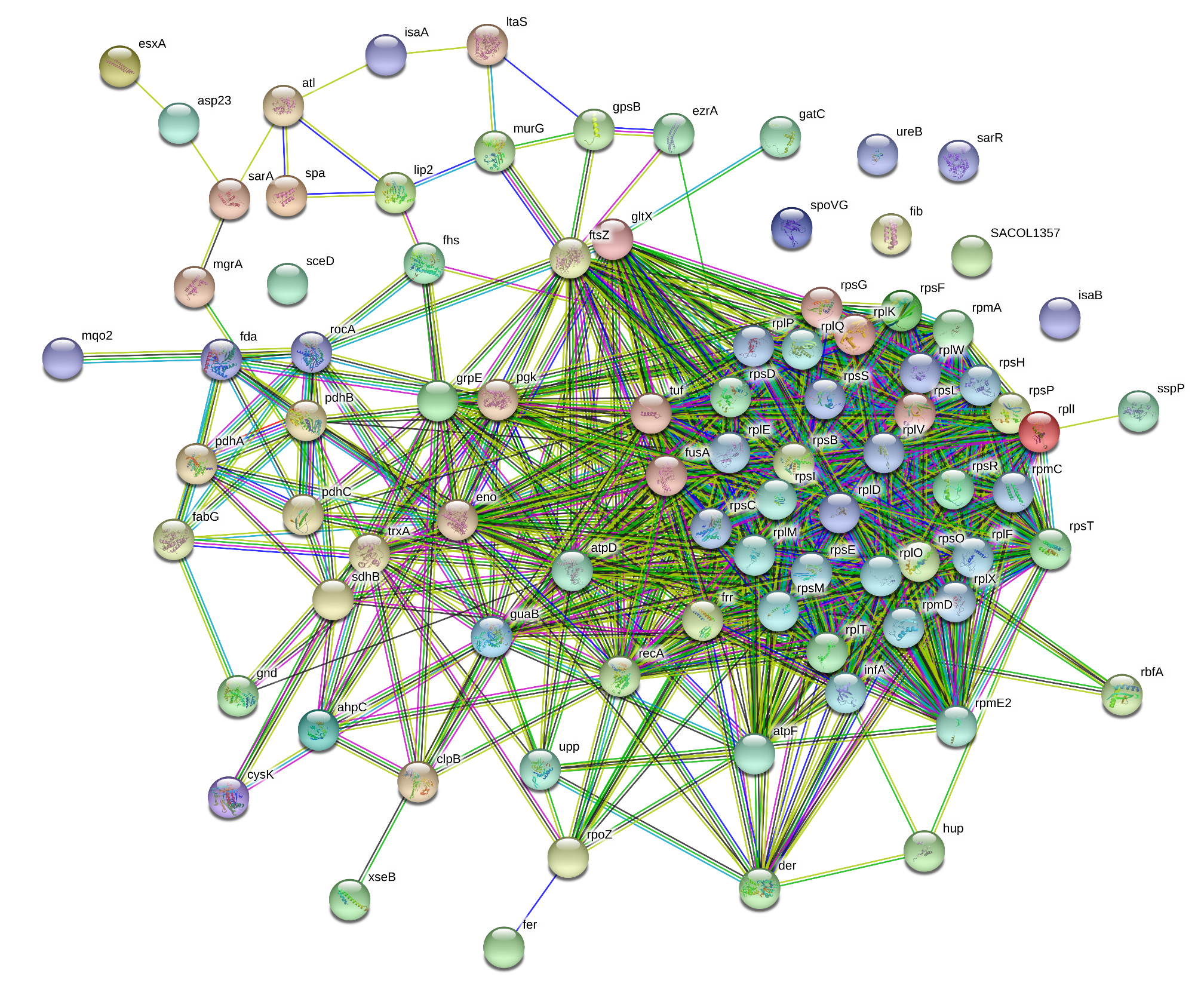


Metabolism/

Ribosomal

Glucose metabolism

ATP-binding

Virulence factors

Virulence factors

Virulence factors

GTP-binding

Lipid catabolism/

Virulence factors

GTP-binding

Virulence factors

Transmembrane transport/ATP Biosynthesis

TCA cycle

Shock protein

Cleavage peptidoglycan

Cell cycle

Cell cycle

Cell cycle

GTP-binding

TCA cycle

DNA-binding

Stress protein
